# Genomic and transcriptomic resources for assassin flies including the complete genome sequence of *Proctacanthus coquilletti* (Insecta: Diptera: Asilidae) and 16 representative transcriptomes

**DOI:** 10.7717/peerj.2951

**Published:** 2017-01-31

**Authors:** Rebecca B. Dikow, Paul B. Frandsen, Mauren Turcatel, Torsten Dikow

**Affiliations:** 1 Office of Research Information Services, Office of the Chief Information Officer, Smithsonian Institution, Washington, D.C., United States of America; 2 Department of Entomology, National Museum of Natural History, Smithsonian Institution, Washington, D.C., United States of America

**Keywords:** Transcriptomics, Asilidae, Draft genome, Genomics, Phylogenomics

## Abstract

A high-quality draft genome for *Proctacanthus coquilletti* (Insecta: Diptera: Asilidae) is presented along with transcriptomes for 16 Diptera species from five families: Asilidae, Apioceridae, Bombyliidae, Mydidae, and Tabanidae. Genome sequencing reveals that *P. coquilletti* has a genome size of approximately 210 Mbp and remarkably low heterozygosity (0.47%) and few repeats (15%). These characteristics helped produce a highly contiguous (N50 = 862 kbp) assembly, particularly given that only a single 2 × 250 bp PCR-free Illumina library was sequenced. A phylogenomic hypothesis is presented based on thousands of putative orthologs across the 16 transcriptomes. Phylogenetic relationships support the sister group relationship of Apioceridae + Mydidae to Asilidae. A time-calibrated phylogeny is also presented, with seven fossil calibration points, which suggests an older age of the split among Apioceridae, Asilidae, and Mydidae (158 mya) and Apioceridae and Mydidae (135 mya) than proposed in the AToL FlyTree project. Future studies will be able to take advantage of the resources presented here in order to produce large scale phylogenomic and evolutionary studies of assassin fly phylogeny, life histories, or venom. The bioinformatics tools and workflow presented here will be useful to others wishing to generate *de novo* genomic resources in species-rich taxa without a closely-related reference genome.

## Introduction

**Figure 1:**
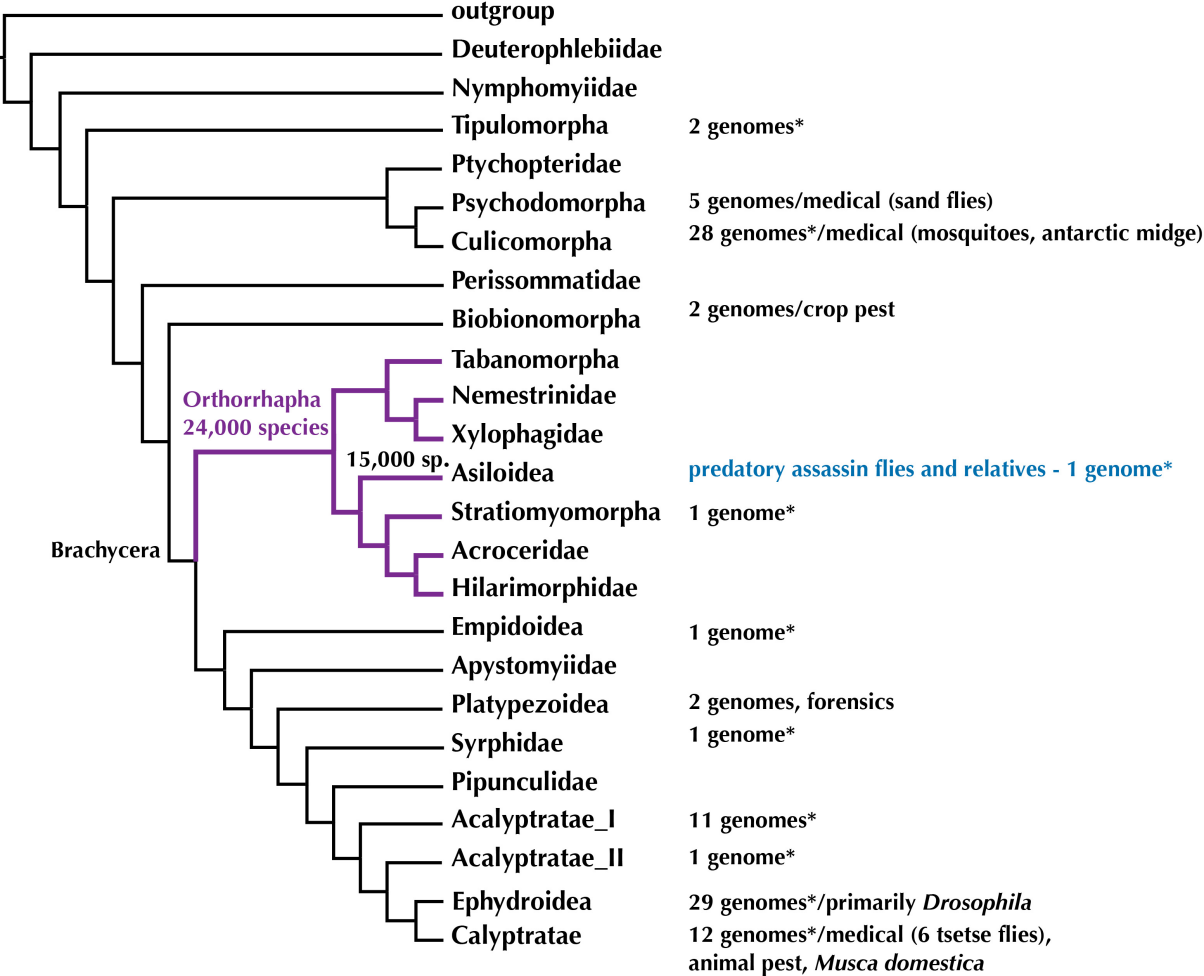
Phylogeny of Diptera (summary tree of hypothesis with higher taxa by Wiegmann et al., 2011) with number of completed genomes and position of Asiloidea. * = includes low-coverage genomes published recently in Vicoso & Bachtrog (2015). Figshare doi: 10.6084/m9.figshare.4056057.

The evolution of genomes within midges, mosquitoes, and flies—Diptera—is better understood than for any other insect order with some 100 whole genomes that have been sequenced and are publicly available. However, the available Diptera genomes are not evenly distributed across this 250 Million year old radiation and skewed towards medically important malaria-transmitting mosquitoes (24 genomes) and species of *Drosophila* used as model organisms in genetic research (29 genomes) (Fig. 1). Here, we provide the first high-quality draft genome and several transcriptomes for orthorrhaphous flies and specifically Asiloidea in the center of the Diptera Tree of Life.

Assassin flies (or robber flies, Diptera: Asilidae) are a diverse group of orthorrhaphous flies with more than 7,500 species known to date (Pape, Blagoderov & Mostovski, 2011). Their common name originates from their predatory behavior in the adult life stage: catching other insects or spiders in flight and injecting their venomous saliva to kill the prey and to liquefy the internal organs to suck out the prey (Dikow, 2009b; Fisher, 2009). Assassin flies have several unique adaptations in proboscis and sucking-pump morphology that enable them to inject venom into their prey and suck out the tissue (Dikow, 2009b). These adaptations and changes in life history from a nectar-feeding ancestor, which is still found in the sister group to Asilidae composed of Apioceridae and Mydidae (Dikow, 2009b; Dikow, 2009a; Trautwein, Wiegmann & Yeates, 2010; Wiegmann et al., 2011), have accelerated their diversification over the past 112 Million years as Apioceridae and Mydidae combined have only 619 described species. The oldest definitive fossils for both Asilidae and Mydidae are Cretaceous in age from the Santana Formation in Brazil (Grimaldi, 1990; Willkommen & Grimaldi, 2007) and Wiegmann et al. (2011) estimate the age of the clade (Asilidae + (Apioceridae + Mydidae)) to be 135 Million years.

### Genomes available for Diptera

To date, out of the 160,000 species of Diptera (Pape, Blagoderov & Mostovski, 2011), complete genomes have been sequenced for 100 species (NCBI as of 04 October 2016). These represent 47% of the insect genomes available at NCBI and are concentrated within the earliest radiation of Diptera including the medically important mosquitoes (Culicidae) and sand flies (Psychodidae) and the higher flies including the model organism *Drosophila* and medically important *Glossina* tsetse flies (Fig. 1).

A recent study by Vicoso & Bachtrog (2015) added some 37 low-coverage genomes for a study on the sex chromosomes of Diptera. While the genome sequencing in this publication was not intended to add draft genomes, the genomes are spread across the Diptera Tree of Life (Fig. 1) and Vicoso & Bachtrog (2015) added two low-coverage (approximately 12×) genomes for Orthorrhapha in the center of fly evolution, i.e., the soldier fly *Hermetia illucens* (Stratiomyomorpha, estimated genome size = 1.3 Gbp, N50 = 2,778 bp) and the assassin fly *Holcocephala fusca* (Asiloidea, estimated genome size = 673 Mbp, N50 = 4,591 bp).

*Aedes*, *Anopheles*, and *Culex* mosquitoes and *Drosophila* vinegar flies shared a common ancestor some 240 Million years ago (mya) (Wiegmann et al., 2011). The most recent common ancestor of mosquitoes and Orthorrhapha likewise lived 240 mya and that of *Drosophila* and Orthorrhapha some 200 mya. Filling a gap in the center of the Diptera Tree of Life (Fig. 1) by providing data on novel, high-quality draft genomes from within Orthorrhapha and Asiloidea will open the opportunity to more meaningfully compare genomes across Diptera. Furthermore, the genomic resources provided here will advance the study of evolutionary history, life history, and the search for the venom of assassin flies.

## Methods

### Specimen source

Adult flies were hand-netted either directly from their resting/perching sites (Apioceridae, Asilidae, Bombyliidae, and Mydidae) or from within a Malaise Trap (Tabanidae) and kept alive in individual vials. They were identified to species, assigned unique identifiers, and either preserved in RNAlater (specimen cut open and placed directly in RNAlater) or liquid N_2_ (specimen alive in individual vial dropped in dry shipper containing liquid N_2_). RNAlater vials were emptied of any liquid before being placed in liquid N_2_-filled tanks in the NMNH Biorepository where all specimens are stored and accessible by their unique specimen identifier (Table 1).

**Table 1:** List of species included in study along with unique specimen identifier of sequenced specimen and preservation method.

Family: subfamily	Species	Specimen identifier	Preservation
Apioceridae	*Apiocera parkeri* Cazier, 1941	USNMENT01136047	liquid N_2_
Asilidae: Asilinae	*Machimus occidentalis* (Hine, 1909)	USNMENT00951022	RNAlater
Asilidae: Asilinae	*Philonicus albiceps* (Meigen, 1820)	USNMENT01027314	RNAlater
Asilidae: Asilinae	*Proctacanthus coquilletti* Hine, 1911	USNMENT01136140	liquid N_2_
Asilidae: Asilinae	*Proctacanthus coquilletti*^a^	USNMENT01136139	liquid N_2_
Asilidae: Asilinae	*Tolmerus atricapillus* (Fallén, 1814)	USNMENT01027313	RNAlater
Asilidae: Brachyrhopalinae	*Nicocles dives* (Loew, 1866)	USNMENT00951000	RNAlater
Asilidae: Dasypogoninae	*Diogmites neoternatus* (Bromley, 1931)	USNMENT00802587	liquid N_2_
Asilidae: Laphriinae	*Laphystia limatula* Coquillett, 1904	USNMENT01136024	liquid N_2_
Asilidae: Stenopogoninae	*Scleropogon duncani* Bromley, 1937	USNMENT01136006	liquid N_2_
Asilidae: Stichopogoninae	*Lasiopogon cinctus* (Fabricius, 1781)	USNMENT00802771	RNAlater
Bombyliidae: Ecliminae	*Thevenetimyia californica* Bigot, 1875	USNMENT00951006	RNAlater
Mydidae: Ectyphinae	*Ectyphyus pinguis* Gerstaecker, 1868	USNMENT01136013	liquid N_2_
Mydidae: Mydinae	*Messiasia californica* (Cole, 1969)	USNMENT01136023	liquid N_2_
Mydidae: Mydinae	*Mydas clavatus* (Drury, 1773)	USNMENT00802763	liquid N_2_
Tabanidae: Pangoniinae	*Fidena pseudoaurimaculata* Lutz, 1909	USNMENT01137217	liquid N_2_
Tabanidae: Tabaninae	*Tabanus discus* Wiedemann, 1828	USNMENT01137218	liquid N_2_

**Notes.**

a denotes specimen for which the genome was sequenced.

### RNA-Seq

Total RNA was extracted from specimens preserved in RNAlater or in liquid N_2_ (see Table 1). A single specimen was used for each extraction. Muscular tissue was extracted from the thorax and cryogenically ground using CryoMill (Retsch, Haan, Germany). Total RNA was isolated using the TRI Reagent Protocol (Sigma-Aldrich, St. Louis, MO, USA) with overnight precipitation, and then quantified using Epoch Microplate Spectrophotometer and Gen5 software (both BioTek, Winooski, VT, USA). For the specimens sequenced using Ion Torrent, the isolation of mRNA was carried out using DynaBeads mRNA DIRECT Kit, and Ion Total RNA-Seq Kit (v2) for Whole Transcriptome Libraries (Thermo Fisher Scientific) was used for library preparation. The BluePippin System (Sage Science, Beverly, MA, USA) was used for selecting fragments of 170–350 bp. For the specimens sequenced using Illumina MiSeq and HiSeq2000, the isolation of mRNA and construction of stranded mRNA-Seq libraries were carried out using KAPA Stranded mRNA-Seq Kit (Kapa Biosystems, Boston, MA, USA) and NEBNext Multiplex Oligos (New England BioLabs, Ipswich, MA, USA). Library fragment size distribution was assessed using High Sensitivity D1000 ScreenTape System (Agilent, Waldbronn, Germany), and the BluePippin System was used for selecting fragments of 180–440 bp. After size selection, a sample of each library was quantified using the KAPA Library Quantification Kit for Illumina platforms, and pooled to 5 nM total concentration for sequencing.

**Figure 2:**
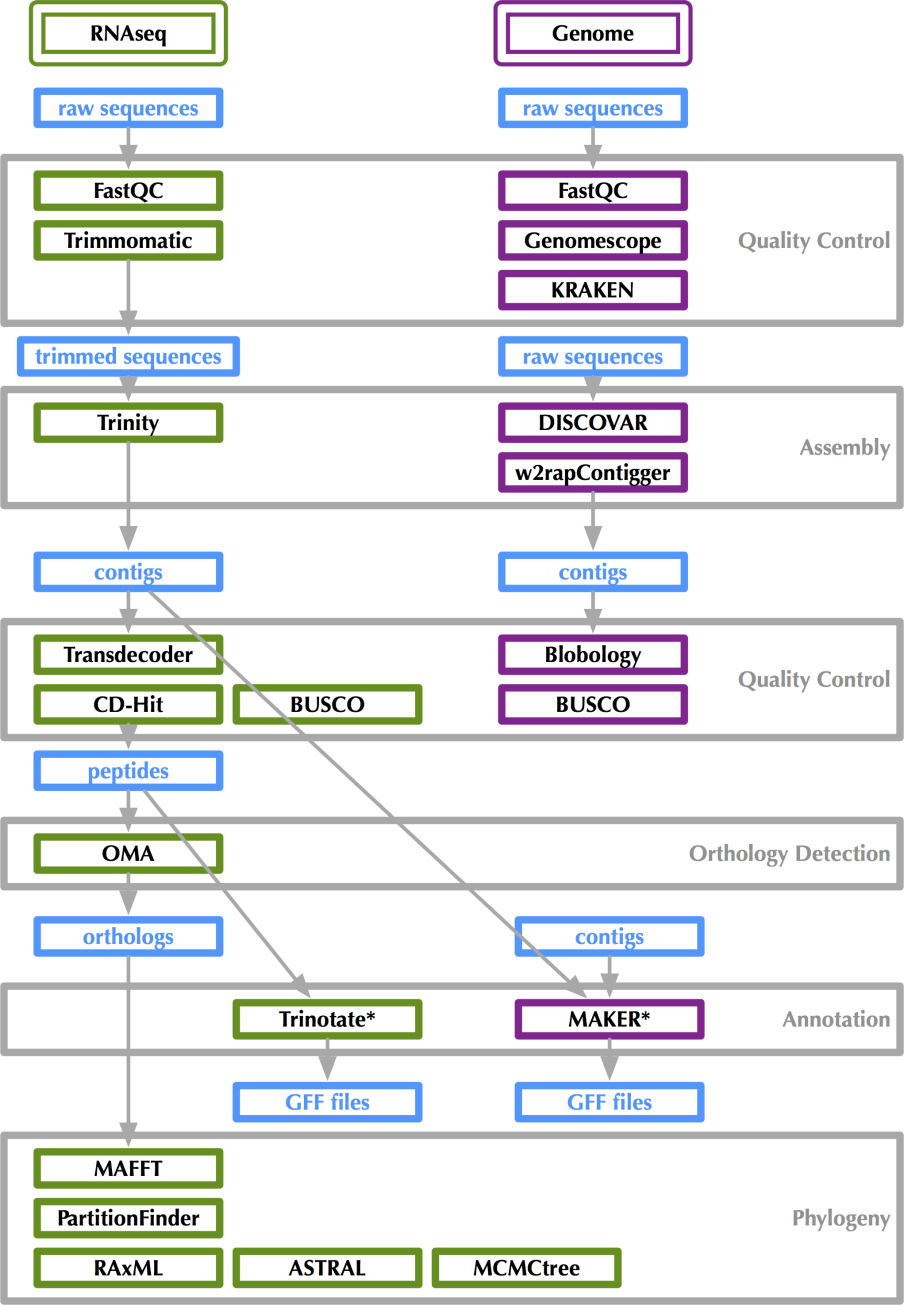
Bioinformatics workflow for transcriptome and genome analysis. Figshare doi: 10.6084/m9.figshare.4056069.

**Figure 3:**
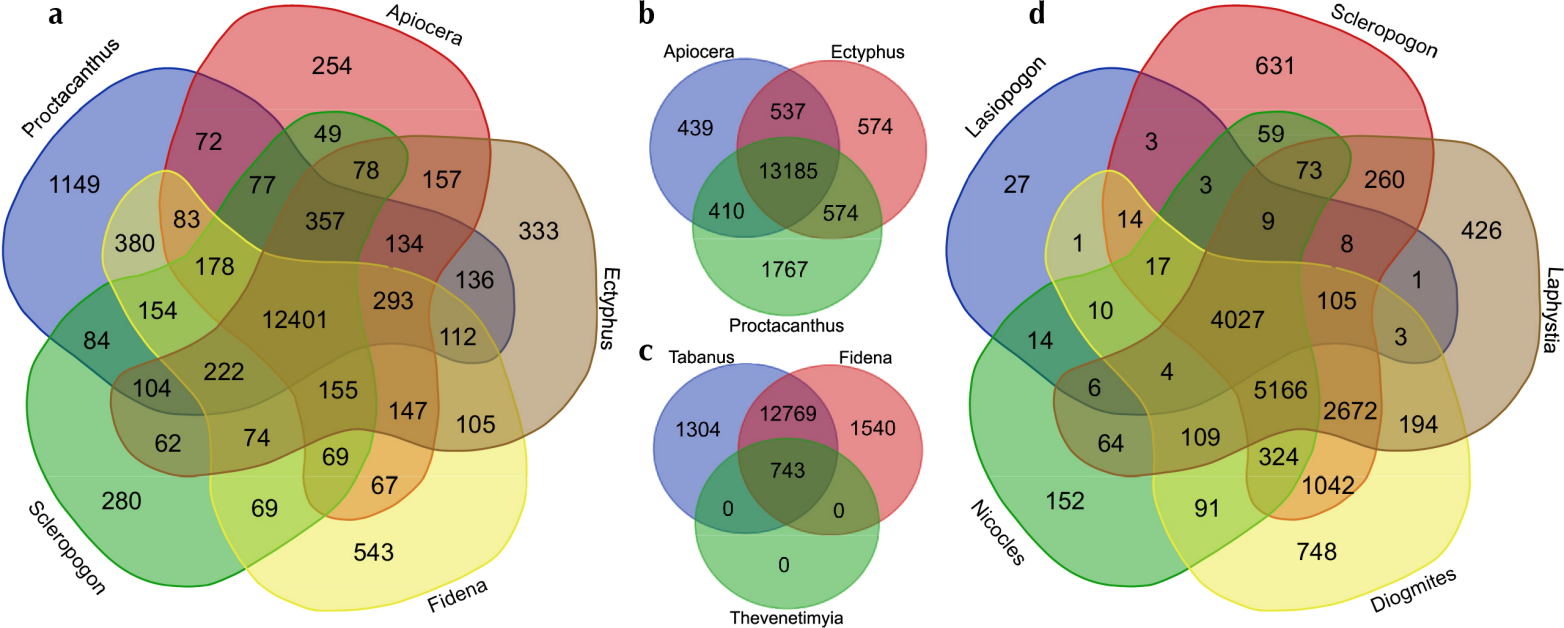
Venn diagrams showing the numbers of GO (Gene Ontology) terms among selected sets of taxa. Visualized at: http://bioinformatics.psb.ugent.be/webtools/Venn/, figshare doi: 10.6084/m9.figshare.4056054.

RNA-Seq bioinformatics workflow is shown in Fig. 2. Raw data as well as assembled transcripts were screened for contamination with KRAKEN (Wood & Salzberg, 2014). RNA-Seq reads were trimmed with Trimmomatic (Bolger, Lohse & Usadel, 2014) and transcripts were assembled in Trinity (Grabherr et al., 2011). Transcriptome “completeness” was estimated using BUSCO (v2.0beta, Simão et al. (2015)) with the “Endopterygota” lineage specific set of 2,442 loci and the -m tran setting. BUSCO assesses completeness with near-universal single copy orthologs selected from OrthoDB (Kriventseva et al., 2015). The 16 sets of assembled transcripts were translated with Transdecoder (Haas & Papanicolao, 2015) under default parameters. Peptides were filtered for redundancy using CD-Hit (v4.6.1, Fu et al. (2012)) specifying a 95% similarity threshold. The Trinotate workflow was used for transcript annotation (Grabherr et al., 2011). Trinotate uses evidence from BLASTx, BLASTp (Altschul et al., 1990), PFAM (Punta et al., 2012), and HMMER (Finn, Clements & Eddy, 2011) to assign GO terms (Ashburner et al., 2000) to transcripts. Venn diagrams showing overlapping sets of GO-terms were generated at: http://bioinformatics.psb.ugent.be/webtools/Venn/ (see Fig. 3).

### Genome sequencing

Genomic DNA was extracted from thoracic muscular tissue and legs of a single specimen of *Proctacanthus coquilletti* preserved in liquid N_2_, using the DNEasy DNA Extraction kit (Qiagen, Hilden, Germany). The sample was quantified using Epoch Microplate Spectrophotometer and Gen5 software, and subsequently pooled to 50 ng/µL concentration. Sequencing took place at Johns Hopkins University Genetic Resources Core Facility’s High Throughput Sequencing Center. A PCR-free library was generated and two lanes of Illumina HiSeq2500 were sequenced to satisfy DISCOVAR recommendations.

**Figure 4:**
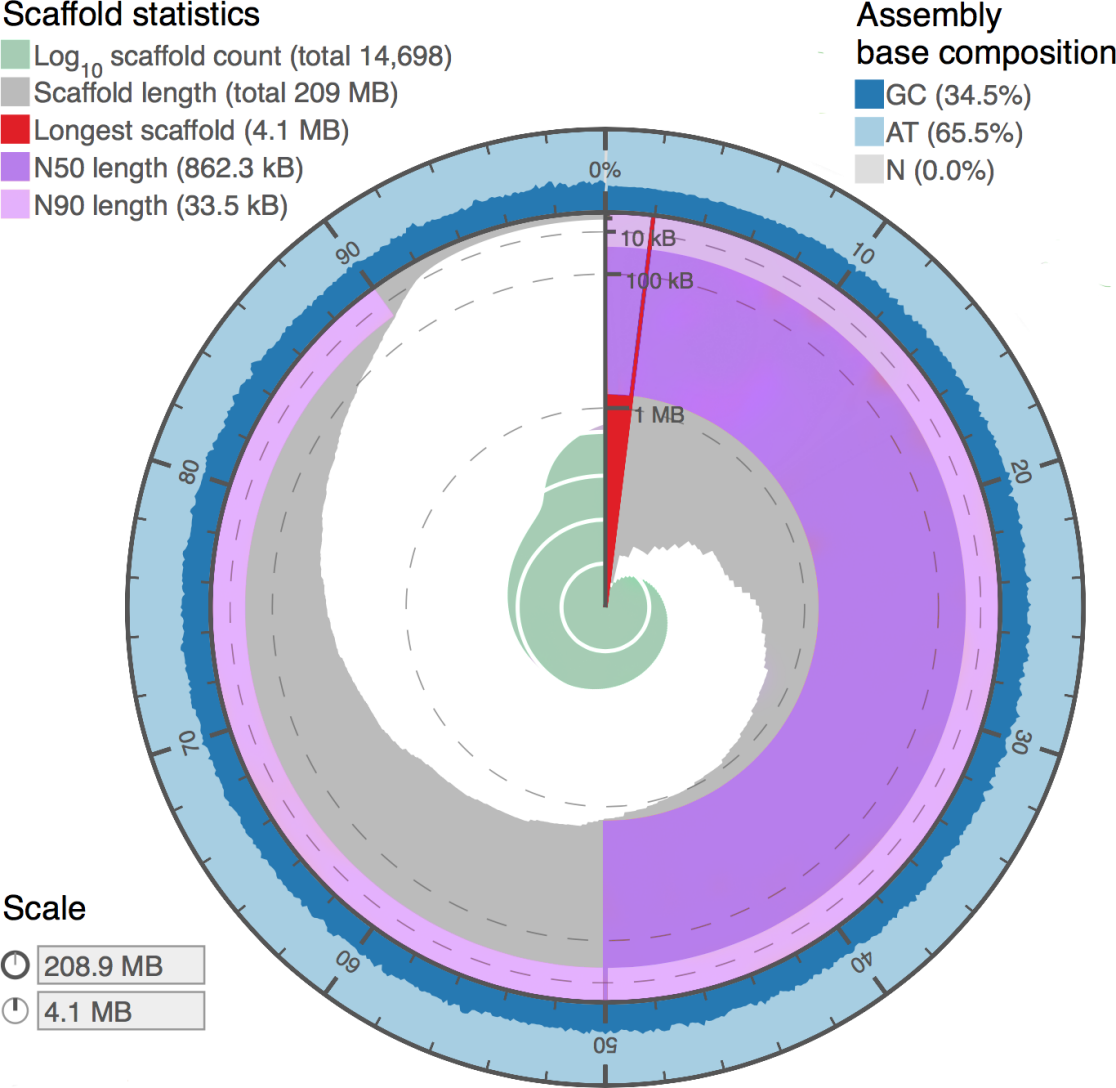
Genome assembly statistics visualization of *Proctacanthus coqilletti de novo* genome (w2rap-contigger 200 kmer assembly, see also Table 5). Visualized at: http://lepbase.org/assembly-statistics/, figshare doi: 10.6084/m9.figshare.4056042.

Genome sequencing bioinformatics workflow is shown in Fig. 2. Genome size, heterozygosity, and repeat content were estimated with raw reads using GenomeScope (Sedlazeck, Nattestad & Schatz, 2016), which uses a kmer histogram generated by JELLYFISH (Marçais & Kingsford, 2011). Raw data as well as assembled contigs were screened for contamination with KRAKEN. Blobtools/Blobology (Kumar et al., 2013) was also used to assess contamination. Sequences were assembled using DISCOVAR *de novo* (Jaffe, 2015) and w2rap-contigger (Clavijo, 2016) with a kmer size of 200 and 260. w2rap-contigger provides performance improvements on DISCOVAR *de novo*, which is no longer being actively developed. Some scaffolding was performed as with 2 × 250 bp reads there is some space between overlap and DISCOVAR *de novo* and w2rap-contigger perform scaffolding internally as shown in Fig. 4. Genome completeness was estimated using BUSCO with the “Endopterygota” lineage specific set of loci and the -m genome setting.

Gene prediction was performed using MAKER (Cantarel et al., 2008), which uses RepeatMasker (Smit, Hubley & Green, 2013), Augustus (Stanke & Waack, 2003), BLAST (Altschul et al., 1990), Exonerate (Slater & Birney, 2005), and SNAP (Korf, 2004). Contigs shorter than 2 kbp were not annotated, as they are too short to produce high-quality evidence. The maximum intron size was set as 10 kbp, which is recommended based on *Drosophila* intron sizes. The Augustus model species used was “fly” and *Drosophila melanogaster* Repbase libraries (Bao, Kojima & Kohany, 2015) were used for RepeatMasker.

### Blobplots

A blobplot was created using blobtools (Kumar et al., 2013, v 0.9.19.5). Prior to generating the blobplot, two steps need to be taken: (1) the raw reads need to be mapped back to the genome to generate an estimate of sequencing coverage and (2) a taxon assignment for each contig needs to be generated by querying the NCBI nucleotide database. Raw reads were mapped using Bowtie 2 (Langmead & Salzberg, 2012, v 2.2.9) and taxon assignments generated using megablast (Altschul et al., 1990; Zhang et al., 2000).

### Phylogenetic trees

Orthology detection was conducted in OMA standalone (v1.0.6, http://omabrowser.org/standalone/) using peptides processed in Transdecoder and CD-Hit. Amino acid alignments on individual resulting orthologs were conducted in MAFFT (Katoh, Asimenos & Toh, 2009). Phylogenetic model selection was performed with PartitionFinderProtein (Lanfear et al., 2012). Gene trees and trees of concatenated, partitioned, data were built in RAxML (raxmlHPC-PTHREADS-SSE3, Stamatakis (2014)). Best tree searches were run 100 times each and rapid bootstrapping was run under the AutoMRE option. ASTRAL (Mirarab et al., 2014) was used to generate a species tree based on individual gene tree topologies.

### Fossil calibrations

Seven fossils ranging in age from 112–45 Million years old (myo) were used to calibrate the time-tree analysis. The maximum age of the root was set to 195 million years (my), an age proposed for the most recent common ancestor of Tabanidae and Asilidae (Wiegmann et al., 2011). MCMCtree, part of the PAML package (Yang, 2007), was used to generate a time-calibrated phylogeny based on the best-scoring RAxML tree.

### Sequence, genome, and analysis data

The raw and assembled sequence data can be accessed under NCBI Umbrella BioProject PRJNA345052. Individual BioProject and BioSample accession numbers are provided in Table 2. The *Proctacanthus coquilletti de novo* genome assembly (w2rap-contigger 200 kmer) can be accessed under NCBI WGS MNCL00000000 and the Genomescope results at: http://qb.cshl.edu/genomescope/analysis.php?code=TRpKdSHytjlB1vBGsPne. Digital copies of visualizations, alignments, and phylogenetic trees can be accessed under a Figshare Collection (doi: 10.6084/m9.figshare.c.3521787, Table S1).

**Table 2:** List of species included in study along with NCBI BioProject, BioSample, and Sequence Read Archive (SRA) numbers for access to raw sequencing reads. Also accessible under NCBI Umbrella BioProject PRJNA345052.

Species	NCBI BioProject	NCBI BioSample	NCBI SRA
*Apiocera parkeri*	PRJNA343825	SAMN05803830	SRR4346321
*Machimus occidentalis*	PRJNA343807	SAMN05802935	SRR4345231
*Philonicus albiceps*	PRJNA343818	SAMN05803661	SRR4365562
*Proctacanthus coquilletti* genome	PRJNA343047	SAMN05772833	SRR4372731
*Proctacanthus coquilletti* transcriptome	PRJNA343047	SAMN05799370	SRR4346725
*Tolmerus atricapillus*	PRJNA343802	SAMN05800340	SRR4346294
*Nicocles dives*	PRJNA343892	SAMN05804943	SRR4345232
*Diogmites neoternatus*	PRJNA343891	SAMN05804928	SRR4345333
*Laphystia limatula*	PRJNA343827	SAMN05803875	SRR4346311
*Scleropogon duncani*	PRJNA343798	SAMN05800191	SRR4346727
*Lasiopogon cinctus*	PRJNA343889	SAMN05804927	SRR4345233
*Thevenetimyia californica*	PRJNA343898	SAMN05804952	SRR4345230
*Ectyphyus pinguis*	PRJNA343820	SAMN05803663	SRR4346320
*Messiasia californica*	PRJNA343822	SAMN05803780	SRR4346726
*Mydas clavatus*	PRJNA343821	SAMN05803778	SRR4345448
*Fidena pseudoaurimaculata*	PRJNA343896	SAMN05804949	SRR4346296
*Tabanus discus*	PRJNA343894	SAMN05804948	SRR4346303

**Table 3:** Summary of RNA-Seq results. BUSCO results are based on a complete set out of 2,442.

Species	Sequencing	Total reads	Total	Total	Transcripts	BUSCO
	platform		RNA	transcripts	with GO	complete
*Apiocera parkeri*	HiSeq2000	143,373,948	1.80	298,313	14,571	1,588
*Machimus occidentalis*	Ion Torrent	2,754,607	29.31	9,330	9,600	52
*Philonicus albiceps*	HiSeq2000	107,425,636	0.32	46,977	15,775	2,212
*Tolmerus atricapillus*	HiSeq2000	108,444,670	0.78	43,915	14,417	2,190
*Proctacanthus coquilletti*	MiSeq	21,978,654	12.46	56,925	15,936	1,933
*Nicocles dives*	Ion Torrent	3,540,783	7.95	10,585	10,452	70
*Diogmites neoternatus*	HiSeq2000	120,499,106	39.14	43,199	14,527	1,951
*Laphystia limatula*	HiSeq2000	60,777,554	3.70	30,019	13,127	607
*Scleropogon duncani*	HiSeq2000	111,276,014	2.80	50,672	14,413	1,693
*Lasiopogon cinctus*	Ion Torrent	2,805,003		1,677	4,252	13
*Thevenetimyia californica*	Ion Torrent	3,050,487	34.60	4,318	743	38
*Ectyphyus pinguis*	MiSeq	29,249,574	9.17	60,424	14,870	1,661
*Messiasia californica*	HiSeq2000	109,427,750	3.20	42,895	14,438	1,329
*Mydas clavatus*	HiSeq2000	90,390,602	1.10	54,643	16,778	1,536
*Fidena pseudoaurimaculata*	MiSeq	32,434,530	16.13	132,246	15,052	1,343
*Tabanus discus*	MiSeq	42,458,502	7.72	96,506	14,816	1,915

## Results and Discussion

### RNA-Seq

RNA-Seq results are shown in Table 3. It is clear that the Ion Torrent platform was the far inferior sequencing platform in terms of total reads, transcripts, and BUSCO recovered loci. We include these four species in the phylogenomic analyses discussed later in spite of the poor wet-lab results because they still provide sufficient homology to generate a phylogenetic hypothesis even though the number of orthologs is small. The MiSeq and HiSeq results are quite comparable, showing that the larger number of reads generated by the HiSeq is not necessary to achieve strong BUSCO results. Four taxa were pooled on each HiSeq lane, and produced approximately three times the raw data of each MiSeq run. For all samples, data were gathered based on a single life-stage, and therefore do not represent the complete transcriptome, which would require larvae and multiple replications. Larvae are almost impossible to find, making this goal unlikely to be achieved. However, for a number of species, *P. albiceps*, *P. coquilletti*, *T. atricapillus*, *T. discus*, *E. pinguis*, in particular, very high-quality sets of transcripts were produced that represent the overwhelming majority of BUSCOs.

The number of GO terms (duplicates removed) in common (overlapping) or unique (not overlapping) for four separate subsets of taxa are summarized in Fig. 3. Figure 3A shows representatives from the major lineages included, i.e., , *Apiocera* (Apioceridae), *Ectyphus* (Mydidae), *Fidena* (Tabanidae), *Proctacanthus* and *Scleropogon* (separate Asilidae clades). Figure 3B shows just one representative from each Asiloid family, Fig. 3C the “outgroup” taxa (Tabanidae and Bombyliidae), and Fig. 3D Asilidae exclusive of the Asilinae clade. Considering that the most recent common ancestor of horse flies (Tabanidae) and asiloid flies (Apioceridae, Asilidae, and Mydidae) existed some 195 mya, the GO term overlap among these taxa is quite large with 12,401 terms in common to the best-sequenced taxa (Fig. 3A). Interestingly, the species selected for genome sequencing, *Proctacanthus coquilletti*, has some 1,149 unique GO terms (Fig. 3A). Among the asiloid flies, some 13,185 shared GO terms were found (Fig. 3B) and here again *Proctacanthus coquilletti* shows a very high number of unique terms (1,767)—the highest in our comparative study. Similarly high unique GO term numbers are found in the sequenced horse flies (Fig. 3C), which might suggest that blood-sucking and predatory flies have a higher number of unique terms compared to nectar-eating flies such as Apioceridae and Mydidae (Fig. 3B) or Bombyliidae (Fig. 3C). The shared GO terms in Fig. 3D for a clade of Asilidae are much lower, which is most likely caused by the reduced number of Ion Torrent reads for *Lasiopogon* and *Nicocles*.

*A. parkeri*, the only species of Apioceridae sequenced, produced more than two times the number of transcripts than any other taxon (Table 3). *A. parkeri* also has the most raw reads, but for other species the number of reads does not show the same relationship to the number of transcripts. *A. parkeri* did not have the highest BUSCO score (i.e., did not produce the most complete single copy orthologs of all the sequenced transcriptomes), but it produced a large number of transcripts with assigned GO terms (14,571), which is the 7th highest number. *A. parkeri* does not have an unusual number of unique GO assignments (compared to the other taxa we sampled, Figs. 3A–3B). *F. pseudoaurimaculata* and *T. discus*, the two species of Tabanidae included, have higher number of transcripts and GO terms. With fewer total transcripts, Mydidae (*Ectyphus pinguis*, *Mydas clavatus*, and *Messiasia californica*, Table 3), the sister group to Apioceridae, reach a high number of GO terms that are with one exception higher than for *Apiocera*. Interestingly, the only included fly with parasitoid larvae, the bee fly *Thevenetimyia*, has by far the lowest number of GO terms (Table 3), however, this transcriptome was sequenced on the Ion Torrent platform. As more species from these taxa are sequenced for transcriptomes and genomes we will gain the ability to investigate whether this pattern will hold and why it might be. Horse flies, mydas flies, and apiocerid flies have very different life histories than assassin flies. Almost all females of horse flies are blood-feeders as adults, while males feed on nectar (Lessard et al., 2013; Morita et al., 2015). Adult flies of Apioceridae and Mydidae are nectar- or honeydew-feeders (Norris, 1936; Paramonov, 1953; Cazier, 1982; Wharton, 1982), if they feed at all.

As a quality check, we ran KRAKEN on all assembled transcripts, which for each species resulted in approximately 0.5% of transcripts having any kmer match to a database of all finished RefSeq bacterial, archaeal, and viral genomes at NCBI. This left us with confidence that we are not including contaminants in our numbers in Table 3.

### Genome sequencing

One HiSeq2500 flow cell (2 lanes) produced 382,575,358 reads. Pre-assembly assessment of kmer distributions in JELLYFISH to produce a histogram, which is then interpreted by GenomeScope, is summarized in Table 4. GenomeScope provides an estimate of genome size of just under 200 Mbp, a very low rate of heterozygosity (appproximately 0.47%), a small percentage of repeats (approximately 15%), and a very low error rate (0.82%). Before submitting *P. coquilletti* for sequencing, we did not have a reliable estimate for any of these parameters, and perhaps could have been successful with a single lane of sequencing, since the genome structure does not appear particularly challenging when compared to many other insect genomes. The low-coverage *Holcocephala fusca* genome (Vicoso & Bachtrog, 2015) is reported to have a genome size of 673 Mbp, more than three times larger than we estimate for *P. coquilletti*. The contig N50 value for *H. fusca* is only 1,778 bp, however, making it a less reliable estimate than the one presented here.

**Table 4:** GenomeScope results (21 kmer). Graphical results available at: http://qb.cshl.edu/genomescope/analysis.php?code=TRpKdSHytjlB1vBGsPne and at figshare doi: 10.6084/m9.figshare.4495940.

Property	Minimum	Maximum
Heterozygosity	0.468619%	0.479918%
Genome Haploid Length	199,195,451 bp	199,343,868 bp
Genome Repeat Length	14,161,640 bp	14,172,191 bp
Genome Unique Length	185,033,812 bp	185,171,677 bp
Model Fit	95.4213%	96.0784%
Read Error Rate	0.820201%	0.820201%

Another positive point about assassin flies beyond their relative genomic simplicity is the large thoracic muscle mass that can be used for DNA and RNA extraction. The gut did not have to be included in the extraction to produce enough DNA, which for a PCR-free library is substantial (3 µg). Since the gut is the source of the most obvious contaminants (meals and gut microbiome), this can be an important factor for those sequencing insect genomes.

### Genome assembly and annotation

Assembly statistics from DISCOVAR *de novo* and w2rap-contigger are summarized in Table 5. Our final assembly was produced by the w2rap-contigger 200 kmer assembly. One of the reasons we chose to try w2rap-contigger was that DISCOVAR produced a much larger than expected genome size estimate. We realized that a set of sequences that are appended to the DISCOVAR *a.lines.fasta* file and are equal to or smaller in length than raw reads and do not represent valid contigs was appended erroneously. DISCOVAR’s own N50 calculator ignores these sequences, and it was only by using our own metadata parser that we found a vastly different N50 value and decided to investigate the assembly output further. We feel that the PCR-free DISCOVAR recipe has produced a very high quality draft genome, particularly given that it is based on a single paired-end library. A plot summarizing the contigs and BUSCO results utilizing the www.lepbase.org assembly statistics tools (Challis, 2016) is shown in Fig. 4 and the number of complete BUSCOs is 2,383, or 97.5%. We chose to remove contigs smaller than 750 bp in all of our assemblies (Table 5) because the combined read length for forward and reverse reads is 500 bp and anything smaller than 750 bp is not likely to be of high quality.

**Table 5:** Assembly results from DISCOVAR *de novo* and w2rap-contigger. BUSCO results are based on a complete set out of 2,442.

Assembler	kmer	N50 (bp)	L50	Longest contig (bp)	Total bp	# contigs	BUSCO complete	GC content
DISCOVAR	200	773,395	75	4,068,162	209,188,750	14,577	2,385	34.50
w2rap-contigger	200	862,345	69	4,070,316	208,912,469	14,698	2,383	34.51
w2rap-contigger	260	379,768	137	2,831,783	198,869,200	6,601	2,378	34.74

The assessment of the contamination of the *P. coquilletti de novo* genome using Blobology (Kumar et al., 2013) reveals that there is very little contamination with the vast majority of hits being arthropods (57.46%) or either unmapped (21.04%) and no-hit (21.19%) (Figs. S1–S2 and Table S2).

Preliminary annotation with MAKER (*Drosophila* reference libraries) produced 10,246 putative genes. *D. melanogaster* has a comparable genome size (164 Mbp compared to 210 Mbp for *P. coquilletti*; NCBI), but more than 17,000 genes (FlyBase.org). We plan to refine our preliminary annotation with more training and manual curation in order to improve our estimate. The *P. coquilletti* MAKER GFF file has been deposited at Figshare (doi: 10.6084/m9.figshare.4055643).

After we finished analyzing both the transcriptomes and genome, and conducted the phylogenetic analyses discussed below, we made a tally of the software programs used to generate these results: 30. This does not include all dependencies, so it is a bit of a conservative estimate. The sheer number of pieces of software in which a researcher using genome-scale data must feel at least conversant is quite large. These data do not lend themselves well to bioinformatics pipelines, either, because there are constantly improvements in existing software that change something about their usage or even new software that is found to be better for certain portions of the workflow and flexibility is key, which can come at the expense of a fairly steep learning curve for researchers just getting their feet wet with genome-scale data. The best way to combat the barrier (whether it is perceived or real) for those who might be interested in developing a set of genomic resources for non-model taxa is to thoroughly document which and how existing software was used (Fig. 2). While it is not possible to exactly recreate one group’s analysis because the computational infrastructure available is undoubtedly different, keeping track of how analyses are done is the first step toward reproducible work. Finally, we know that because tools are constantly improving, all current genomic resources are really just drafts that will be improved upon as we improve the software and databases upon which our results rely.

**Figure 5:**
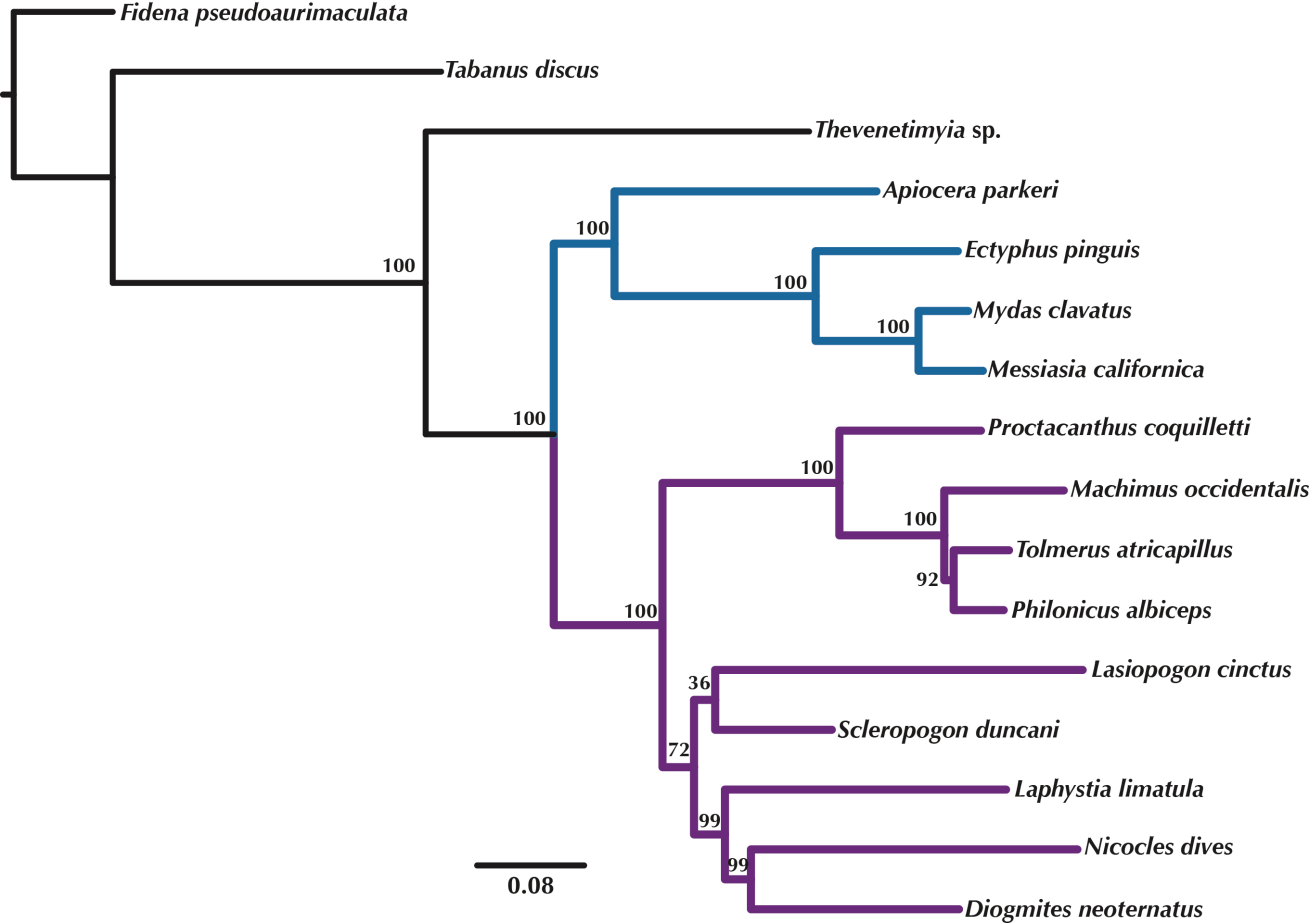
Maximum Likelihood tree of the concatenated matrix of orthologous transcripts predicted in OMA from RAxML. Included are orthologs represented by four or more species and Bootstrap values are shown above the branches. Purple branches = Asilidae, blue branches = Apioceridae + Mydidae. Figshare doi: 10.6084/m9.figshare.4055910.

### Phylogenetic trees

OMA produced 9,080 putative orthologs shared among four or more species. There were only a small number of loci found in all taxa because of the four Ion Torrent transcriptomes are of subpar quality. While we plan to resequence these on an Illumina platform in the future, they did produce enough data to be placed convincingly in a phylogenetic framework, which will be used to design exon capture probes in order to sample hundreds more species for which we have DNA but no RNA-quality specimens. The concatenated alignment length was 4,936,984 amino acid residues. Individual locus alignments as well as the concatenated alignment and best partitioning scheme from PartitionFinderProtein have been deposited at Figshare (concatenated alignment doi: 10.6084/m9.figshare.4055622, PartitionFinderProtein doi: 10.6084/m9.figshare.4055814).

**Figure 6:**
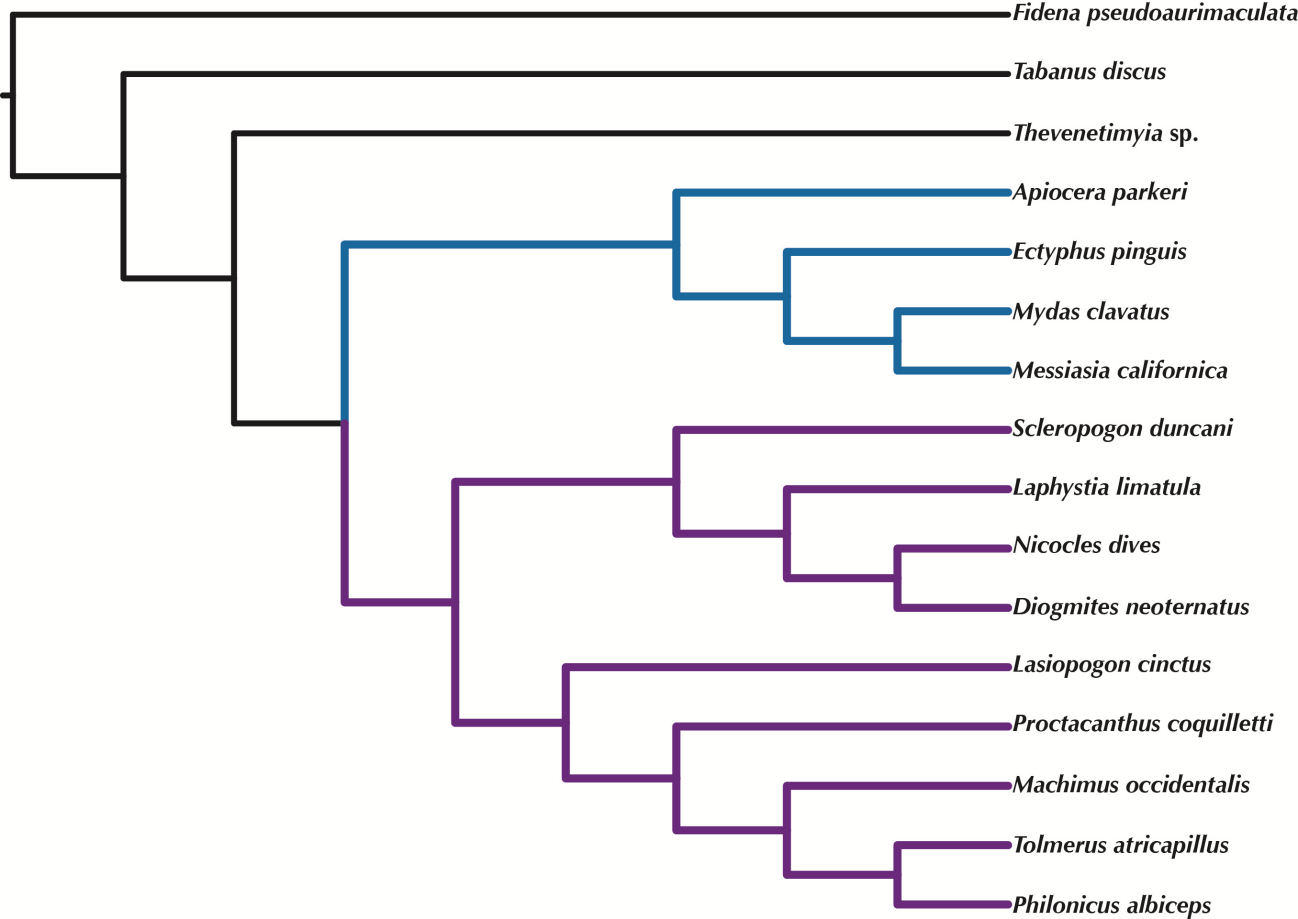
ASTRAL tree based on RAxML best trees of the concatenated matrix for all orthologs with four or more species. Purple branches = Asilidae, blue branches = Apioceridae + Mydidae. Figshare doi: 10.6084/m9.figshare.4055781.

Orthologous loci obtained from the transcriptomes were used to construct phylogenetic trees (Figs. 5–6) for the 16 taxa. While the small taxon sampling is not sufficient to provide new insights into the relationships within Mydidae (3 species included) or Asilidae (10 species), some comments on the higher-level relationships among and within families can be made. Both hypotheses (Figs. 5–6) support the position of Bombyliidae (*Thevenetimyia californica*) being more closely related to the other Asiloidea taxa Apioceridae, Asilidae, and Mydidae than to Tabanidae (Tabanomorpha, see also Fig. 1). A taxon Apioceridae plus Mydidae is supported as monophyletic and as the sister-group to Asilidae as previously proposed (Dikow, 2009b; Dikow, 2009a; Trautwein, Wiegmann & Yeates, 2010; Wiegmann et al., 2011). The relationships within Mydidae support the monophyly of the subfamily Mydinae with the two included genera *Messiasia* and *Mydas*. Within Asilidae, the four included Asilinae genera form a monophylum (*Machimus*, *Philonicus*, *Proctacanthus*, and *Tolmerus*) with *Proctacanthus* recovered as sister-group to the remaining three genera. The clade (*Laphystia* (*Diogmites* + *Nicocles*)) is recovered in both analyses, but with a different sister-group. The placement of the genus *Lasiopogon*, which has the fewest data available (Table 3), differs in the analyses. The tree based on concatenated loci (Fig. 5) places *Lasiopogon* as sister-group to *Scleropogon*, which is the least supported clade (bootstra*p* value of 36), and both genera are placed together as sister-group to (*Laphystia* (*Diogmites* + *Nicocles*)). In the ASTRAL analysis (Fig. 6), *Lasiopogon* is placed as sister-group to the Asilinae. The Laphriinae, here represented by *Laphystia*, has not been recovered as the earliest divergence within Asilidae has postulated by Dikow (2009b) and Dikow (2009a).

### Time-calibrated tree

†*Araripogon axelrodi* and †*Cretomydas santanensis*, representing the oldest, definitive Asilidae (Grimaldi, 1990) and Mydidae (Willkommen & Grimaldi, 2007) fossils, respectively, were placed as sister group to the remaining taxa from these families. †*Burmapogon bruckschi*, a 100 myo Burmese amber fossil (Dikow & Grimaldi, 2014), was placed as sister-group to *Lasiopogon* (see discussion of placement in Dikow & Grimaldi (2014)). Four species of Asilidae are known from Baltic amber with an age of 45 my (Evenhuis, 1994) and a study of 25 newly discovered amber specimens (T Dikow, 2015, unpublished data) adds three additional species. In total, four Baltic amber assassin flies (two previously described and two yet undescribed) are included as they can be sufficiently well-placed. Based on the most recent phylogeny of Asilidae (Dikow, 2009b), †*Asilus klebsi* was placed as sister-group to *Tolmerus* + *Machimus* + *Philonicus*, a yet undescribed Asilinae genus and species with an ovipositor similar to that of extant *Promachus* (Asilinae: Apocleini) as sister-group to the included four Asilinae species, the Laphriinae: Atomosiini †*Protoloewinella keilbachi* as sister-group to *Laphystia tolandi*, and an undescribed genus and species of Stenopogoninae as sister group to *Scleropogon duncani*.

**Figure 7:**
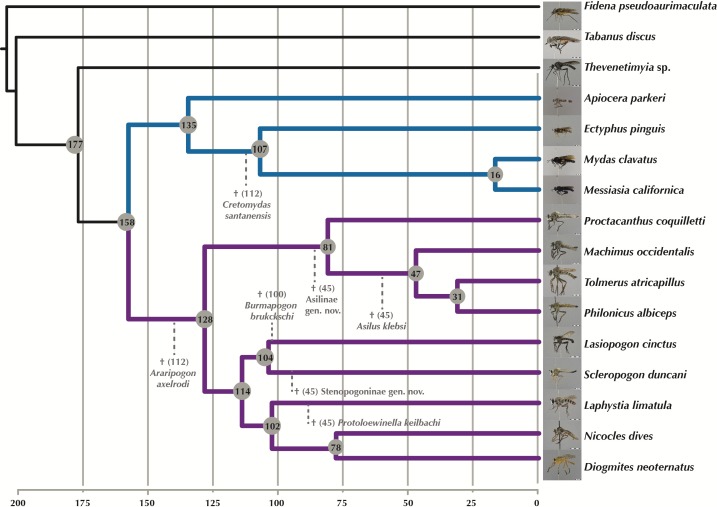
Time-calibrated phylogeny generated with MCMC tree. Fossil calibration points indicated by dotted lines and hypothesized divergence dates shown at the nodes. Purple branches = Asilidae, blue branches = Apioceridae + Mydidae. Images of flies represented in the USNM collection taken by B Wingert, M Gisonda, and T Dikow. Figshare doi: 10.6084/m9.figshare.4055997.

With the inclusion of seven Cretaceous and Tertiary fossils, we provide the first time-calibrated tree for ages within Apioceridae, Asilidae, and Mydidae (Fig. 7). The limited taxon sampling in our analysis prohibits a detailed discussion of clade ages, nonetheless the results provide a first view of the earliest divergences within these taxa. The split between Apioceridae + Mydidae and Asilidae is postulated to have occurred about 158 Million years ago (mya), which is approximately 25 my earlier than hypothesized by Wiegmann et al. (2011). Likewise, the split between Apioceridae and Mydidae is here postulated to be 135 mya, 15 my earlier than the age estimated by Wiegmann et al. (2011). Within the included Mydidae, the split between Ectyphinae and Mydinae is postulated to have occurred 107 mya, which is supported by the placement of †*Cretomydas* (112 myo) before the Ectyphinae/Mydinae divergence in a morphological phylogenetic study on the family (T Dikow, 2015, unpublished data). Based on our analysis, the earliest divergence within Asilidae occurred 128 mya and that of the four included Asilinae genera 81 mya. The remaining five subfamilies diverged 114 mya, which is supported by the placement of †*Burmapogon* (100 myo) within this clade.

## Supplemental Information

10.7717/peerj.2951/supp-1 Figure S1 Blobplot of read coverage for contamination assessment in *Proctacanthus coquilletti de novo* genome (w2rap-contigger 200 kmer assembly, see also Table 7) Figshare doi: 10.6084/m9.figshare.4292912.

10.7717/peerj.2951/supp-2 Figure S2 Blobplot of contamination assessment in *Proctacanthus coquilletti de novo* genome Blobplots rely on four items of information: read coverage, taxon assignments of each contig, GC content, and contig length. Each ”blob” represents a single contig and the size of the blob is proportional to the number of nucleotides in that contig. The colors represent the taxon assignment. Red represents ”Arthropoda”, which is the taxon that we would expect the majority of contigs to be assigned to. The y-axis represents coverage and the height of a blob on this axis represents the amount of coverage, which was estimated from raw reads mapped back to that particular contig. The x-axis represents GC content. Frequently, diverse organisms will have different GC contents. This is one way of visualizing whether contigs exist that have vastly different GC content and might not be a part of the genome being visualized. The two histograms along the axes represent coverage (y-axis) and GC content (x-axis). The y-axis on each of these histograms represents the total number of nucleotides in kilobases within each bin, which is calculated from the sum of the lengths of the contigs within that particular bin.

10.7717/peerj.2951/supp-3 Table S1 Digital copies of visualizations, alignments, and phylogenetic trees deposited at Figshare (also accessible under Figshare Collection doi:10.6084/m9.figshare.c.3521787)

10.7717/peerj.2951/supp-4 Table S2 Blopblot data of contamination assessment in *Proctacanthus coquilletti de novo* genome (w2rap-contigger 200 kmer assembly) from which Fig. 5 and Supplementary Fig. 9 were generated

## Additional Information and Declarations

### DNA Deposition

The following information was supplied regarding the deposition of DNA sequences: The raw sequences of the genome and transcriptomes are accessible via the NCBI Umbrella BioProject PRJNA345052. The genome assembly is accessible via NCBI accession number MNCL00000000.

### Data Availability

The following information was supplied regarding data availability: Dikow, Rebecca; Frandsen, Paul; Turcatel, Mauren; Dikow, Torsten (2017): Dikow et al. Genomic and transcriptomic resources for assassin flies. figshare.
https://dx.doi.org/10.6084/m9.figshare.c.3521787.
