# Supplementary figures and images for: Genomic and transcriptomic resources for assassin flies including the complete genome sequence of Proctacanthus coquilletti (Insecta: Diptera: Asilidae) and 16 representative transcriptomes
Source: PeerJ. 2017 Jan 31;5:e2951. doi: 10.7717/peerj.2951 (PMC5289110; doi:10.7717/peerj.2951)

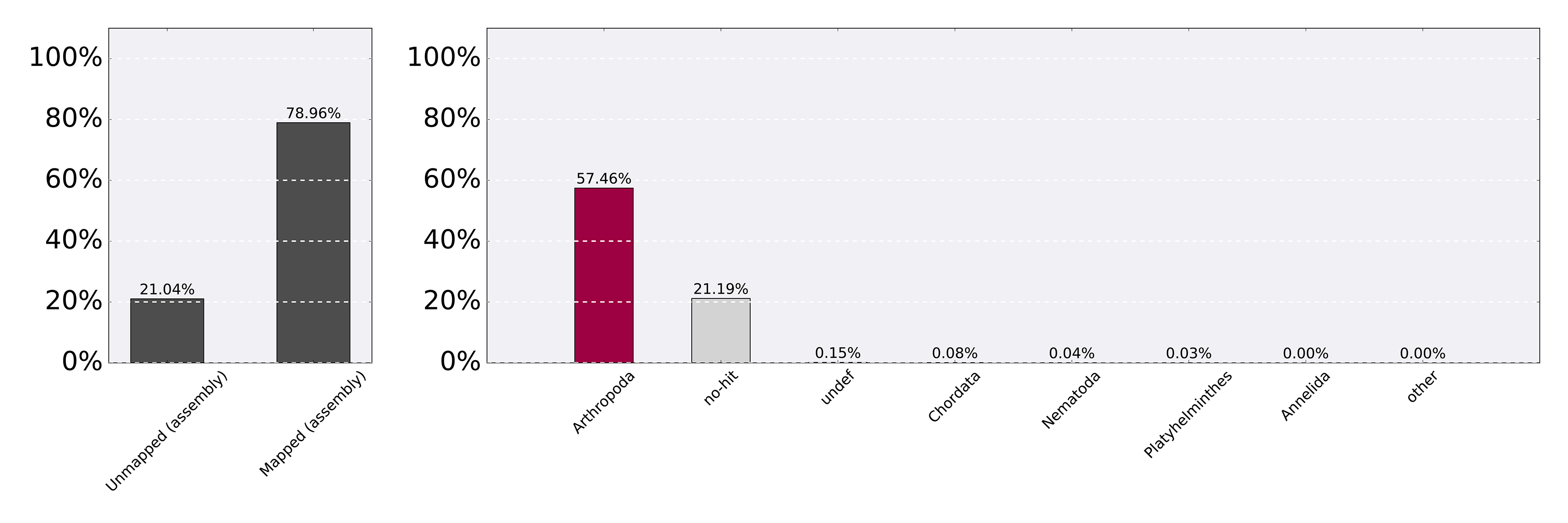

Supplement: Figure S1 — Figshare doi: 10.6084/m9.figshare.4292912. [file peerj-05-2951-s001.png]
